# Is obesity a risk factor in cancer patients with COVID-19?

**DOI:** 10.2217/fon-2021-0676

**Published:** 2021-07-13

**Authors:** Robin Park, Elizabeth Wulff-Burchfield, Weijing Sun, Anup Kasi

**Affiliations:** ^1^Department of Medicine, MetroWest Medical Center/Tufts University School of Medicine, Framingham, MA 01702, USA; ^2^Department of Medicine, Kansas University Cancer Center, Division of Medical Oncology, Kansas City, KS 66205, USA

**Keywords:** COVID-19, obesity, risk determinant

Coronavirus disease 2019 (COVID-19) has caused unprecedented morbidity and mortality in cancer patients worldwide and has resulted in countless delays and missed opportunities in cancer screenings, diagnoses and treatments; an estimated 10,000 excess deaths will result just from delays in the screening and treatment of colorectal and breast cancer in the USA [1]. Given that an accurate risk assessment of cancer patients with COVID-19 remains crucial for patient counseling, vaccine prioritization and patient triage, numerous studies have examined the determinants of increased morbidity and mortality attributable to COVID-19 in cancer patients. Of note, patient baseline characteristics associated with increased mortality include male sex, advanced age (>65 or 70), smoking status, performance status (Eastern Co-operative Oncology Group ≥2), underlying cardiovascular disease and hypertension [2]. These variables are also consistently demonstrated to increase the risk of morbidity and mortality associated with COVID-19 in the general, unselected patient population [3,4]. However, despite most risk determinants being largely similar between the cancer patient and general patient population, obesity interestingly stands out as one that is not.

## Obesity is associated with increased morbidity & mortality in patients with COVID-19 in the general population

Obesity has been frequently and consistently cited as a risk factor for worse outcomes in the setting of COVID-19 in the general patient population. A multicenter, prospective cohort study conducted in the UK that included 20,133 hospitalized patients with COVID-19 demonstrated that obesity was an independent risk factor for in-hospital mortality (hazard ratio [HR]: 1.33; 95% CI: 1.19–1.49) based on a multivariable adjusted analysis [5]. Similarly, a multicenter retrospective cohort study conducted in the USA that included 7606 ambulatory patients who were hospitalized for COVID-19 showed that obesity was associated with a higher risk of mechanical ventilation and/or in-hospital death. In this particular study, the risk association showed a dose–response relationship whereby the severity of obesity was associated with a numerically higher HR of the adverse outcomes [6]. These results are in keeping with the study conducted by the CDC, which perhaps is one of the largest cohort studies to date. The data used were based on the Premier Healthcare Database and comprised a total of 148,494 patients who received emergency department or inpatient care who had an ICD-10 code indicating a diagnosis of COVID-19. Indeed, the results showed that overweight or obesity (based on BMI) was associated with increased risk of mechanical ventilation and death [7]. Of note, these findings are also in line with a recent systematic review and meta-analysis of 75 observational studies that showed obese patients with COVID-19 were at increased risks of hospitalization, ICU admission, mechanical ventilation and mortality compared with their non-obese counterparts [8]. Taken together, the evidence strongly supports obesity as a risk determinant of worse outcomes in COVID-19 in the general patient population.

## Obesity is not associated with increased morbidity & mortality in cancer patients with COVID-19

In contrast, studies in cancer patients have not identified obesity as a determinant of adverse outcomes in COVID-19. In a single-center, prospective cohort study conducted in France of 178 mixed ambulatory and hospital inpatients, obesity was not associated with increased risk of clinical worsening or overall survival (HR: 1.16; 95% CI: 0.36–3.69) in a univariable Cox proportional model [9]. Similarly, a single-center, retrospective cohort studies in the USA of 309 cancer patients with COVID-19 showed that obesity was not associated with severe or critical illness as defined by the presence of tachypnea, hypoxemia, ICU admission, organ failure or death (HR 0.95; 95% CI: 0.64–1.40). A multivariate analysis after adjusting for age, sex, smoking status, comorbidities and performance status consistently showed that obesity was still not associated with severe illness (HR 0.96; 95% CI: 0.62–1.47) [10]. Additionally, the global, multicenter, retrospective COVID-19 and Cancer Consortium cohort study of 928 cancer patients with COVID-19 showed that obesity was not associated with increased 30-day all-cause mortality (odds ratio [OR]: 0.84; 95% CI: 0.50–1.41), the results of which remained consistent after adjusting for age, sex and smoking status (OR: 0.99; 95% CI: 0.58–1.71) [11]. The results of these studies are consistent with a meta-analysis of three retrospective cohort studies of 2117 mixed ambulatory and hospital cancer inpatients that show obesity is not associated with increased risk of all-cause mortality (OR: 0.92; 95% CI: 0.66–1.28). The meta-analysis included studies of good quality and showed low heterogeneity and no significant small-sample publication bias [12].

Similarly, cohort studies of hematologic cancer patients also demonstrate that obesity is not a relevant risk factor for adverse outcomes. In a small, retrospective, case–control study conducted in Spain (n = 39) comprising only patients with hematologic cancer, conducted with inpatient and outpatient data, there was no significant difference between the mean BMI between survivors and non-survivors (p = 0.163) [13]. Similarly, in a retrospective cohort study in the UK also of hematologic cancer patients using inpatient and outpatient data, there was no difference between the mean BMI of survivors and non-survivors (OR: 0.33; 95% CI: 0.09–1.25) [14]. Thus obesity (based on BMI) does not appear to be a significant risk factor for worse outcomes in cancer patients with COVID-19.

## Possible explanations for the discrepancy in risk determinants between patients with & without cancer

An epidemiologic study of the H1N1 virus, responsible for the influenza pandemic of 2009, showed that obesity was an independent risk factor for increased mortality [15]. Additionally, a systematic review and meta-analysis of MERS-CoV infections showed that the prevalence of infection was higher in individuals with obesity compared with their non-obese counterparts [16]. These results are consistent with the finding that obesity is associated with increased morbidity and mortality in patients with COVID-19 in the general population. Several hypotheses have been proposed to explain how obesity increases morbidity and mortality in patients with COVID-19 in the general population, including reduced baseline respiratory function due to body habitus, immune dysregulation due to dysmetabolism, and the additional risk conferred by obesity-associated comorbidities such as diabetes, hypertension and other cardiovascular diseases [17]. Moreover, the abundant expression of ACE-2 receptors on human adipocytes suggests the potential for adipose tissue to function as a viral reservoir for SARS-CoV-2 [18].

On the other hand, an epidemiologic phenomenon has been described in which individuals with obesity paradoxically have lower mortality associated with either acute or chronic illness compared with their non-obese counterparts. This phenomenon, which has been dubbed the ‘obesity paradox’, has been described in epidemiologic studies involving patients with pneumonia [19]. Another cohort study found that short-term mortality in patients hospitalized with infections was lower in overweight and obese patients compared with their normal-weight counterparts after adjusting for comorbidities [20]. Similar phenomena have also been described in the literature associated with chronic diseases such as end-stage renal disease, chronic obstructive pulmonary disease and venous thromboembolism, as well as cancer [21–23]. The leading hypothesis for this phenomenon points to the greater metabolic reserve represented by the abundant adipose tissue, which enables the patient to withstand the course of severe acute or chronic illness, as an explanation.

Taken together, these findings raise the possibility that obesity exerts a protective effect in the setting of comorbid cancer to counterbalance the increased susceptibility it confers in the setting of COVID-19. Furthermore, the same observation can be extrapolated to other comorbidities that have been associated with the obesity paradox; further research is warranted to determine whether obesity has a greater protective effect in certain comorbidities. Consequently, these observations complicate risk determination in patients with COVID-19 because obesity, which is a prevalent and easily assessed risk factor, has confounding variables and is not applicable across all patients.

Notably, there are limitations of using BMI measurements as a proxy for obesity and adiposity; the use of BMI is a caveat that should be taken into account when interpreting the relationship between obesity and clinical outcomes in epidemiologic studies, because body composition can vary greatly among individuals of different ethnicities and ages. Future studies should involve the assessment of more specific body composition metrics, such as muscle mass and subcutaneous and visceral fat mass, to provide further insight [24]. Indeed, a large-scale study dedicated to assessing the impact of obesity and related comorbidities is warranted to adequately dissect the complex relationship between obesity and COVID-19 in cancer patients.

## Conclusion & future perspective

In contrast to the general patient population with COVID-19, obesity does not appear to be a determinant of increased morbidity and mortality associated with COVID-19 in cancer patients. Future studies are warranted to prospectively evaluate the association between anthropometric variables as well as more sophisticated measures of body composition and clinical outcomes in cancer patients with COVID-19 and to determine the potential biological explanation underlying this discrepancy. An accurate assessment of determinants of risk in cancer patients with COVID-19 will be crucial going forward in the optimal, evidence-based management of cancer patients.
